# Beyond the Individual: A Scoping Review of Group and Community Dimensions of Youth Empowerment

**DOI:** 10.3390/bs16060866

**Published:** 2026-05-29

**Authors:** Ruben-David Fernández Carrasco, Gisela Carrillo Bestagno, David Martínez Salguero, Moisés Carmona Monferrer

**Affiliations:** Research Group on Interaction and Social Change, Department of Social and Quantitative Psychology, University of Barcelona, 08035 Barcelona, Spain; rubendavidfernandez@ub.edu (R.-D.F.C.); gcarribe8@alumnes.ub.edu (G.C.B.);

**Keywords:** youth empowerment, psychological empowerment, group empowerment, community empowerment, psychosocial intervention, adolescence, participatory approaches, scoping review

## Abstract

This scoping review examines how youth empowerment is addressed beyond the psychological level, with a focus on group and community dimensions. Although empowerment theory conceptualises empowerment as a multi-level process, research has predominantly focused on individual outcomes. Following PRISMA-ScR guidelines, a systematic search was conducted in Scopus and Web of Science. This search identified 32 empirical studies involving adolescents aged 10 to 18, focusing on psychosocial interventions and outcomes beyond psychological empowerment. The studies were conducted across diverse contexts and mainly employed qualitative and mixed methods designs. The findings show that, at the group level, empowerment is associated with social support, collective learning and participation in decision-making. At the community level, it is reflected in civic engagement, co-design and shifts in power relations and public recognition. However, most studies continue to prioritise psychological outcomes. Groups and communities are primarily treated as contexts rather than as units of empowerment. These results indicate that group and community dimensions remain underdeveloped and highlight the need for approaches that better capture empowerment as a multi-level and relational process.

## 1. Introduction and Theoretical Framework

Youth empowerment focuses on promoting young people’s capacities and mastery in their personal and collective development. It emphasises autonomy and the ability to understand and play a meaningful role in their social environment (Mouchrek & Benson, 2023; Úcar Martínez et al., 2017). This construct has gained increasing relevance in both academic and policy contexts. This is reflected in international frameworks such as the European Union Youth Strategy 2019–2027 (Council of the European Union, 2018).

Within community psychology, empowerment is a central theoretical and practical framework. It is defined as the process through which individuals, organisations and communities gain control over their lives and goals (Rappaport, 1984). Empowerment is understood both as a process and as an outcome. As a process, it involves actions, activities and the creation of empowering structures. As an outcome, it involves the acquisition of knowledge, skills and resources (Rappaport, 1984; Zimmerman, 2000). This dual nature poses challenges for its evaluation. Empowerment processes are highly sensitive to contextual conditions and group-specific needs.

These contextual and developmental considerations are particularly relevant in the case of youth. Youth empowerment differs from adult empowerment in that it must be adapted to young people’s developmental stage. It enables them to acquire capacities to make decisions about their lives and influence their environments (Mouchrek & Benson, 2023; Úcar Martínez et al., 2017; Zimmerman, 2000). This perspective reflects a shift from deficit-oriented approaches focused on prevention and control. It moves toward strengths-based approaches that recognise young people as active agents with valuable contributions to society (Jennings et al., 2006; Khan, 2022; Small & Memmo, 2004).

The literature on youth empowerment encompasses multiple models and conceptualisations, including the Adolescent Empowerment Cycle, the Positive Youth Development and Empowerment Program Model, the Transactional Partnering Model and the Empowerment Education Model (Úcar Martínez et al., 2017). Despite this diversity, common elements include participation, dialogue, support and capacity-building through educational processes. Jennings et al. (2006) synthesised the critical social theory of youth empowerment. They identified key dimensions such as safe and inclusive spaces, meaningful participation, power-sharing between youth and adults, critical reflection on social processes and opportunities for collective action. A wide range of participatory methodologies have been used to promote youth empowerment. These include participatory action research, photovoice, youth leadership programmes and creative and community-based approaches (Soler-Masó et al., 2025). This diversity reflects a field in expansion. However, important gaps remain, particularly in relation to group and community dimensions of empowerment (Soler-Masó et al., 2025).

From a theoretical perspective, empowerment comprises three interrelated levels: individual, group and community (Rappaport, 1984; Zimmerman, 2000). The individual level, often referred to as psychological empowerment, includes intrapersonal, interactional and behavioural components. The intrapersonal component comprises a sense of competence and control over oneself, as well as the ability to influence the environment. The interactional component refers to the use of analytical skills to engage with the sociopolitical environment. The behavioural component involves taking actions to exert control and participate. Christens (2012) additionally highlights a relational component. This includes the development of interpersonal processes based on collaboration, mutual support and facilitating the empowerment of others.

The group level of empowerment refers to the creation or availability of empowering groups or organisations. These groups provide opportunities for people to gain control over their lives. It also refers to empowered groups or organisations that achieve their goals and have sociopolitical influence and participation. Lastly, the community level encompasses all existing or newly created empowering and empowered groups or organisations within a social environment. It also includes the interrelationships between them. These levels are dynamically interconnected. Empowered individuals contribute to the development of empowering and empowered organisations and communities, and vice versa (Zimmerman, 2000).

Despite this multi-level conceptualisation, the literature has predominantly focused on psychological empowerment, both in adult and youth populations (Christens, 2023; Diaz & Paceley, 2024; Lardier et al., 2024; Mouchrek & Benson, 2023). Several explanations have been proposed. These include the relative ease of measuring individual change, the difficulty of capturing group and community processes empirically, and the influence of funding structures that prioritise individual-level outcomes (Christens, 2023; Perkins et al., 2023; Úcar Martínez et al., 2017). From a critical perspective, this tendency has also been linked to individualising frameworks. These frameworks obscure structural and collective dimensions of empowerment (Khan, 2022). The group and community levels, due to their open-ended definitions, require a broader scope of analysis. This may involve the collection of longitudinal data. It may also require promoting the continuity of groups or organisations beyond the punctual implementation of an intervention. This is necessary to assess their long-term success and their integration within the community.

This limitation is particularly evident in research with adolescents. Adolescence represents a developmental stage in which young people progressively acquire autonomy, social participation and cognitive capacities for leadership (Thulin et al., 2022). However, studies focusing on this age group continue to prioritise psychological outcomes and often rely on adult-mediated processes (Cargo et al., 2003; Diaz & Paceley, 2024; Mouchrek & Benson, 2023). These processes may limit the autonomy of young people to create and sustain groups and coalitions led by the teenagers themselves. As a result, the potential to link individual capacities with collective and community processes remains insufficiently explored. This is despite its relevance for fostering meaningful youth empowerment (Jennings et al., 2006; Soler-Masó et al., 2025).

In response to these gaps, this study presents a scoping review of the scientific literature on youth empowerment that explores this construct beyond psychological empowerment. It includes studies that report on psychosocial interventions involving adolescent participants.

While previous reviews have addressed youth empowerment in specific domains—such as mental health, substance use prevention or environmental engagement (Cyril et al., 2016; Adu et al., 2022; Valdez et al., 2020; Kennedy et al., 2019)—or through specific tools or approaches (Chong et al., 2022), they have largely focused on psychological outcomes or related constructs. In this review, empowerment is treated as the central theoretical construct rather than as a general synonym for youth participation, civic engagement, youth organizing or collective action. These concepts are closely related to empowerment, but they are not equivalent to it. From a community psychology perspective, empowerment is understood as a multi-level process involving psychological, group or organisational, and community dimensions (Rappaport, 1984; Zimmerman, 2000). Therefore, the review focuses on studies that explicitly engage with empowerment or closely related constructs within this theoretical tradition. Following the PRISMA Extension for Scoping Reviews (PRISMA-ScR) guidelines (Tricco et al., 2018), this study aims to answer the following research question: how is youth empowerment at the group and community levels reported in the available scientific literature?

## 2. Method

A systematic review (SR) is defined as a structured approach to synthesising scientific literature using explicit, transparent and reproducible methods, with the aim of identifying what is known about a topic, how knowledge has been generated and what gaps remain (Gough et al., 2012). This scoping review was retrospectively registered in the Open Science Framework (OSF) https://doi.org/10.17605/OSF.IO/6UATR.

The present study adopts a scoping review design, a type of knowledge synthesis that allows for the systematic mapping of key concepts, types of evidence and research gaps in a given field (Tricco et al., 2018). This approach is particularly suitable for emerging and heterogeneous areas of research such as youth empowerment.

In line with the exploratory purpose of scoping reviews, no formal methodological quality appraisal tool was applied to the included studies (Tricco et al., 2018). However, several indirect quality criteria were considered during study selection and interpretation. These included publication in peer-reviewed journals indexed in Scopus or Web of Science, clarity in the description of the intervention and methodological procedures, and relevance to the research question.

Differences in methodological designs and levels of evidence were also taken into account during synthesis and interpretation. Consequently, findings were interpreted with caution, particularly in relation to the predominance of qualitative studies and the limited availability of longitudinal evidence.

**Figure 1:**
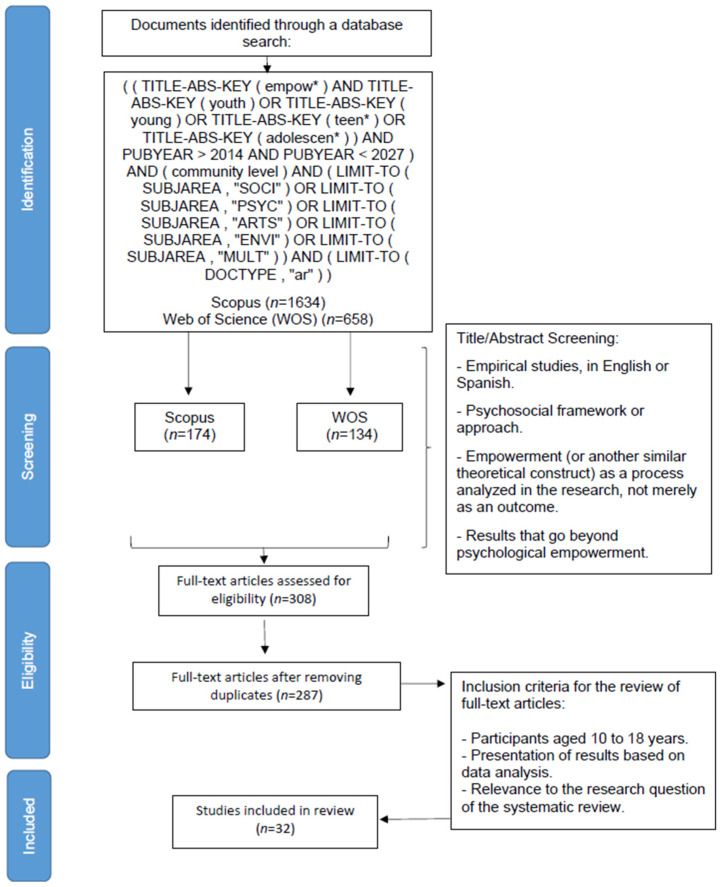
PRISMA flow diagram illustrating the search strategy and article selection process.

The review process was conducted following the PRISMA Extension for Scoping Reviews (PRISMA-ScR) guidelines (Tricco et al., 2018). The procedures included identification, screening and in-depth review of primary studies. The search strategy and selection process are illustrated in Figure 1.

### 2.1. Identification of Primary Studies

Searches were conducted in December 2025 in two electronic databases: Scopus and Web of Science. The same search string was applied in both databases. Given the exploratory nature of scoping reviews, they do not necessarily require a quality assessment of the included studies. This is because the evidence compiled in scoping reviews is not usually limited to randomized clinical trials or observational studies (Lopez-Cortes et al., 2022). Nevertheless, this review used the exclusive search of indexed databases as an indirect quality criterion. Scopus (Elsevier, 2026) indexes journals based on a clearly defined selection policy and an internationally recognized selection committee of experts. Every scientific journal indexed in this database meets the criterion of publishing peer-reviewed articles. Web of Science indexes journals that meet criteria for quality, editorial rigor, and impact (Universidad de Córdoba, 2025). 

Initially, the following search string was used, yielding a total of 5448 documents in Scopus and 13,681 in Web of Science. Upon reviewing the titles and abstracts, it became apparent that the search string was too broad, meaning that most of the documents did not meet the criterion of being a study that went beyond psychological empowerment.

Original search string: (TITLE-ABS-KEY (empow*) AND TITLE-ABS-KEY (youth) OR TITLE-ABS-KEY (young) OR TITLE-ABS-KEY (teen*)) AND PUBYEAR > 2014 AND PUBYEAR < 2027 AND (LIMIT-TO (DOCTYPE, “ar”)) AND (LIMIT-TO (SUBJAREA, “SOCI”) OR LIMIT-TO (SUBJAREA, “PSYC”) OR LIMIT-TO (SUBJAREA, “ARTS”) OR LIMIT-TO (SUBJAREA, “ENVI”) OR LIMIT-TO (SUBJAREA,”MULT”)).

Given the above, the documents identified by the original search query were further filtered using an additional criterion: the community level. This decision was theoretically grounded in the community psychology tradition of empowerment research. In this tradition, empowerment is not limited to individual change but is conceptualised as a multi-level process involving psychological, organisational or group, and community levels (Rappaport, 1984; Zimmerman, 2000). The criterion “community level” was therefore used to identify studies more likely to report empowerment processes beyond psychological empowerment.

Related terms such as collective action, civic engagement, youth organizing and organisational processes were not incorporated as independent search domains. This was a deliberate scope decision. Including these terms would have broadened the review toward the wider fields of youth participation, activism and civic studies. The aim of this review was more specific: to examine how the empowerment literature itself reports group and community dimensions of youth empowerment. Using this refined search string, the databases returned a total of 1634 documents in Scopus and 658 in Web of Science. 

Refined search string: TITLE-ABS-KEY (empow*) AND (youth OR young OR teen* OR adolescen*) AND (community level) AND PUBYEAR > 2014 AND PUBYEAR < 2027 AND (LIMIT-TO (SUBJAREA, “SOCI”) OR LIMIT-TO (SUBJAREA, “PSYC”) OR LIMIT-TO (SUBJAREA, “ARTS”) OR LIMIT-TO (SUBJAREA, “ENVI”) OR LIMIT-TO (SUBJAREA, “MULT”)) AND (LIMIT-TO (DOCTYPE, “ar”)).

### 2.2. Screening

The records retrieved from both databases (*n* = 2292) were screened based on titles and abstracts using the following eligibility criteria: (a) empirical studies including a psychosocial intervention involving participants; (b) a psychosocial framework; (c) empowerment (or a related construct) addressed as a process rather than solely as an outcome; and (d) reported results extending beyond psychological empowerment.

A total of 308 studies met the initial eligibility criteria. After removing duplicates (*n* = 21), a sample of 287 studies was retained for further review.

During screening, studies were assessed not only for the presence of participatory or collective practices, but also for their conceptual alignment with empowerment as a psychosocial and multi-level process. This distinction was important because many studies address youth participation, civic engagement or collective action without framing these processes as empowerment. Such studies were outside the scope of this review unless they explicitly contributed to understanding empowerment beyond the psychological level.

### 2.3. Full-Text Review and Selection of the Final Sample

An in-depth review of the 287 eligible studies was conducted by a research team of four investigators. The inclusion criteria for full-text review were: (a) core of the participants’ age ranged between 10 and 18 years; (b) results based on empirical data; and (c) relevance to the research question of the review. With regard to criterion (a), although the review focused on studies involving adolescents aged 10 to 18, some studies included broader or slightly narrower age ranges. In these cases, studies were retained only when the adolescent population represented the core target group of the intervention or when the findings specifically referred to participants within or closely aligned with the selected age range.

During the full-text review, particular attention was paid to whether the reported empowerment processes and outcomes could be meaningfully interpreted as corresponding to adolescent participants. Studies in which findings could not be analytically distinguished from substantially older populations were excluded from the final sample.

Two researchers independently reviewed 143 and 144 studies, respectively, and made preliminary inclusion decisions. The first reviewer selected 43 studies, while the second selected 33 studies, resulting in a total of 76 potentially relevant articles. A subsequent triangulation process was conducted by two additional researchers, who reviewed the preliminary decisions and identified 32 studies for inclusion in the final sample (*n* = 32), as presented in Table 1. Disagreements among researchers were discussed in team meetings as part of the study’s iterative process, with the primary focus being on how each study’s findings contributed to answering the research question.

The most frequent reasons for exclusion at this stage (*n* = 44) were related to study design (e.g., absence of a psychosocial intervention), sample characteristics (e.g., inability to distinguish the target age range), and focus of analysis (e.g., exclusive focus on psychological empowerment or lack of alignment between the conceptualisation of empowerment and the intervention described). Specifically, the criterion for reporting a psychosocial intervention was based on the need for studies to describe an intervention whose general or specific objective was to achieve youth empowerment. This is because our research question sought to understand how empowerment processes that go beyond the individual level are reported; therefore, it was not appropriate to include studies that assessed empowerment without considering the psychosocial processes required for empowerment to occur. 

### 2.4. Data Analysis

The analysis was guided by the theoretical distinction between psychological, group or organisational, and community levels of empowerment proposed by Zimmerman (2000). During coding, findings were classified according to the primary level of empowerment addressed in each study.

Individual-level processes referred mainly to personal capacities, self-efficacy and individual agency. Group-level processes included relational dynamics such as mutual support, collective learning, shared decision-making and collective action within groups or organisations. Community-level processes referred to participation in civic or institutional spaces, inter-organisational relationships, influence on community decision-making and transformations in public recognition or power relations.

Coding decisions were discussed iteratively among the research team through triangulation meetings in order to strengthen analytical consistency and resolve ambiguities between levels.

The 32 articles comprising the final sample were analyzed using a content analysis approach. First, the research team familiarized itself with the evidence by repeatedly reviewing the studies. Second, a detailed characterization of the studies was conducted, taking into account year of publication, research objectives and design, participants, and data collection and analysis, in order to identify patterns across the included studies. Third, a detailed analysis was conducted of the main findings related to empowerment reported by the studies. At this stage, particular emphasis was placed on findings that go beyond psychological empowerment to map the available knowledge and answer the research question posed. 

In addition to the characterization of the studies, types of psychosocial interventions, and research designs, the analytical procedure made it possible to identify key dimensions of empowerment at the group and community levels. At the group level, the following were identified as key themes: building social support ties and a sense of belonging; collective learning and shared meaning-making; and collaborative action and participation in decision-making within projects. At the community empowerment level, the following were identified as key themes: Civic participation, co-design, and co-governance; and Transformation of power relations and public legitimacy.

## 3. Results

The scoping review process resulted in a final sample of 32 articles. The selected studies represent a heterogeneous body of research addressing psychosocial interventions aimed at promoting youth empowerment, with diversity in geographical contexts, methodologies, participant profiles and conceptual approaches. Overall, the studies reflect a field that is theoretically and methodologically plural, with increasing emphasis on participatory approaches and relational processes.

The results are presented in four sections: (1) characteristics of the studies; (2) types of psychosocial interventions; (3) study designs; and (4) main findings related to group and community empowerment.

### 3.1. Characteristics of the Studies

The 32 studies were conducted across diverse geographical contexts, including North America—particularly the United States (Abraczinskas & Zarrett, 2020; Brown et al., 2015; Cardarelli et al., 2021; Case, 2017; Dill, 2015; Ellington et al., 2023; Goodkind et al., 2020; Godfrey & Grayman, 2014; Horwitz & Mares, 2025; McKay et al., 2022; Ruhr & Fowler, 2022; Wagaman et al., 2023; Wellman et al., 2025)—Latin America (González et al., 2022; Montes et al., 2022; Moreno Álvarez, 2025; Massó Guijarro, 2018; Monteiro et al., 2015; Trott et al., 2020), Europe (Cicognani et al., 2015; Buelens et al., 2017; Favre et al., 2025; Scholtes-Bos et al., 2025; Ferrer-Fons et al., 2022), Africa (Bastable et al., 2023; Forbes-Genade & van Niekerk, 2018; Zuma et al., 2022), Asia (To et al., 2020; Farahzadi et al., 2025; Hillel Lavian, 2018) and Oceania (Wenitong-Schrieber et al., 2024; Lasczik et al., 2025).

This geographical diversity indicates that youth empowerment interventions are implemented across heterogeneous socio-political contexts.

Regarding participants, the studies included diverse profiles such as racialised youth and ethnic minorities (Brown et al., 2015; Dill, 2015; Godfrey & Grayman, 2014; Goodkind et al., 2020; Wagaman et al., 2023; Ellington et al., 2023; Horwitz & Mares, 2025), migrants and refugees (Farahzadi et al., 2025; Goodkind et al., 2020; Ferrer-Fons et al., 2022; Favre et al., 2025), Indigenous youth (Wenitong-Schrieber et al., 2024; Trott et al., 2020), adolescents in contexts of poverty or social exclusion (Bastable et al., 2023; Forbes-Genade & van Niekerk, 2018; Monteiro et al., 2015; Massó Guijarro, 2018; Case, 2017; González et al., 2022; Montes et al., 2022; Moreno Álvarez, 2025; Hillel Lavian, 2018; Zuma et al., 2022), and adolescents with disabilities (Bastable et al., 2023; Lasczik et al., 2025). The focus on some profiles is geographically specific: poverty and social exclusion are the main targets of interventions in Latin America and Africa, while racialized youth and ethnic minorities stand out in North America.

With regard to age range, most of the selected studies included samples within the range established as an inclusion criterion (Abraczinskas & Zarrett, 2020; Brown et al., 2015; Buelens et al., 2017; Cardarelli et al., 2021; Case, 2017; Ellington et al., 2023; Farahzadi et al., 2025; Forbes-Genade & van Niekerk, 2018; Godfrey & Grayman, 2014; González et al., 2022; Goodkind et al., 2020; Hillel Lavian, 2018; Lasczik et al., 2025; McKay et al., 2022; Montes et al., 2022; Trott et al., 2020; Wellman et al., 2025); some of the selected studies using slightly broader ranges (Bastable et al., 2023; Cicognani et al., 2015; Dill, 2015; Favre et al., 2025; Ferrer-Fons et al., 2022; Horwitz & Mares, 2025; Massó Guijarro, 2018; Monteiro et al., 2015; Ruhr & Fowler, 2022; Scholtes-Bos et al., 2025; To et al., 2020; Wagaman et al., 2023; Wenitong-Schrieber et al., 2024; Zuma et al., 2022) and one study did not report a specific age range, other than the “youth” category (Moreno Álvarez, 2025). 

### 3.2. Types of Psychosocial Interventions

#### 3.2.1. Settings and Contexts of Implementation

Interventions were implemented in diverse settings, including schools and youth programmes (Abraczinskas & Zarrett, 2020; González et al., 2022; To et al., 2020; Wellman et al., 2025), community organisations and NGOs (Ellington et al., 2023; Wenitong-Schrieber et al., 2024; Forbes-Genade & van Niekerk, 2018), public spaces (Montes et al., 2022; Horwitz & Mares, 2025; Moreno Álvarez, 2025), and artistic–cultural environments such as theatre and audiovisual production (Massó Guijarro, 2018; Lasczik et al., 2025; Favre et al., 2025; Hillel Lavian, 2018; Trott et al., 2020).

#### 3.2.2. Intervention Strategies

The studies reported diverse strategies, including participatory methodologies (Abraczinskas & Zarrett, 2020; Dill, 2015; Wagaman et al., 2023), creative and arts-based approaches (Hillel Lavian, 2018; Massó Guijarro, 2018; Favre et al., 2025; Trott et al., 2020), and civic engagement initiatives such as environmental or community projects (González et al., 2022; Horwitz & Mares, 2025; Moreno Álvarez, 2025; Wellman et al., 2025). Intervention strategies are evenly distributed across groups or continents; only civic engagement initiatives are more specific to North America and Latin America. 

Some studies also highlighted the relevance of youth–adult power dynamics (Brown et al., 2015; Cardarelli et al., 2021; To et al., 2020).

### 3.3. Study Designs

The studies showed methodological diversity, including qualitative (*n* = 20), mixed-methods (*n* = 8) and quantitative designs (*n* = 4).

Qualitative studies included ethnographies, participatory action research and creative methodologies (Dill, 2015; Massó Guijarro, 2018; Forbes-Genade & van Niekerk, 2018; Wagaman et al., 2023; Hillel Lavian, 2018; Trott et al., 2020; Ellington et al., 2023; Lasczik et al., 2025; Wenitong-Schrieber et al., 2024).

Mixed-methods studies combined surveys with qualitative data (Abraczinskas & Zarrett, 2020; Goodkind et al., 2020; McKay et al., 2022; Wellman et al., 2025).

Quantitative studies examined variables such as empowerment, participation and self-efficacy (Godfrey & Grayman, 2014; Cicognani et al., 2015; To et al., 2020; Farahzadi et al., 2025).

### 3.4. Group and Community Empowerment

#### 3.4.1. Group-Level Empowerment

Group-level empowerment was reflected in processes of social support and belonging (Ellington et al., 2023; Favre et al., 2025; Ferrer-Fons et al., 2022; Horwitz & Mares, 2025; Lasczik et al., 2025; Ruhr & Fowler, 2022), collective learning and meaning-making (Abraczinskas & Zarrett, 2020; Bastable et al., 2023; González et al., 2022; Trott et al., 2020; Wagaman et al., 2023), and participation in decision-making processes (Cardarelli et al., 2021; Case, 2017; Montes et al., 2022; Moreno Álvarez, 2025).

##### Building Social Support Ties and a Sense of Belonging

Six of the selected articles (Ellington et al., 2023; Favre et al., 2025; Ferrer-Fons et al., 2022; Horwitz & Mares, 2025; Lasczik et al., 2025; Ruhr & Fowler, 2022) show that participation in stable group settings enables networks of support and belonging that sustain youth agency and mutual care. The emergence of a sense of group belonging, described by Buelens et al. (2017); Cicognani et al. (2015); Dill (2015); Hillel Lavian (2018); Massó Guijarro (2018); Montes et al. (2022) and Scholtes-Bos et al. (2025), constitutes an essential affective foundation for group empowerment. Ellington et al. (2023) demonstrate that these ties take shape through relationships of continuity, accompaniment, and emotional safety, fostering sustained engagement in the project, well-being, and the construction of collective identities. Similarly, Goodkind et al. (2020) highlight that mutual recognition and solidarity among Afro-descendant girls support processes of resistance in response to shared experiences of racialization and adult centrism, generating forms of collective support and belonging. This aligns with the findings of Forbes-Genade and van Niekerk (2018), who identify collective identity, solidarity, and mutual support as relational dimensions of empowerment among adolescent girls from an informal settlement in South Africa characterized by poverty.

##### Collective Learning and Shared Meaning-Making

Several studies, including Abraczinskas and Zarrett (2020), Bastable et al. (2023), Buelens et al. (2017), Cardarelli et al. (2021), Case (2017), Dill (2015), Ellington et al. (2023), Favre et al. (2025), Ferrer-Fons et al. (2022), Forbes-Genade and van Niekerk (2018), González et al. (2022), Hillel Lavian (2018), Horwitz and Mares (2025), Lasczik et al. (2025), Massó Guijarro (2018), Monteiro et al. (2015), Montes et al. (2022), Moreno Álvarez (2025), Ruhr and Fowler (2022), Scholtes-Bos et al. (2025), Trott et al. (2020), Wagaman et al. (2023), and Wenitong-Schrieber et al. (2024), provide evidence that groups function as privileged spaces for the collective production of knowledge.

Within these spaces, young people interpret situated experiences, construct counter-hegemonic narratives, and develop shared critical analyses. The group becomes a setting where strategies for critically understanding the social world are learned. Methodologies such as photovoice, as well as the creation of audiovisual materials or poetry, enable the translation of situated experiences into critical reflection and public representations, as shown by Ellington et al. (2023); Hillel Lavian (2018); Dill (2015); Favre et al. (2025); Lasczik et al. (2025); Monteiro et al. (2015); Montes et al. (2022); and Trott et al. (2020).

The collective nature of these practices transforms problems initially experienced as individual into shared social diagnoses, a mechanism documented in at least 20 studies, including Case (2017), Forbes-Genade and van Niekerk (2018), Ferrer-Fons et al. (2022), Ruhr and Fowler (2022), Cardarelli et al. (2021), and Wagaman et al. (2023). In this same vein, Goodkind et al. (2020) emphasize that identifying shared experiences of racial injustice enabled participants to “name” forms of oppression and develop a critical understanding of them.

##### Collaborative Action and Participation in Decision-Making Within Projects

Group empowerment is also expressed in young people’s capacity to act collectively and participate in decision-making processes within their groups and projects, for example through the allocation of responsibilities, the organization of activities, the facilitation of meetings, and shared decision-making with adult facilitators.

This dimension is particularly evident in 22 of the 32 studies, especially those employing participatory methodologies such as YPAR, PAR, photovoice, artistic co-creation, and citizen science models (Abraczinskas & Zarrett, 2020; Bastable et al., 2023; Buelens et al., 2017; Cardarelli et al., 2021; Case, 2017; Dill, 2015; Ellington et al., 2023; Favre et al., 2025; Ferrer-Fons et al., 2022; Forbes-Genade & van Niekerk, 2018; González et al., 2022; Goodkind et al., 2020; Hillel Lavian, 2018; Horwitz & Mares, 2025; Lasczik et al., 2025; Massó Guijarro, 2018; Monteiro et al., 2015; Montes et al., 2022; Moreno Álvarez, 2025; Scholtes-Bos et al., 2025; Trott et al., 2020; Wenitong-Schrieber et al., 2024). For instance, both Abraczinskas and Zarrett (2020) and Cardarelli et al. (2021) show that group empowerment is expressed through the active participation of young people in collaborative, action-oriented processes, in which they identify relevant problems, deliberate collectively, negotiate priorities, share power with adult actors, and co-design proposals with practical impact.

Overall, the selected studies show that collective action and participation in decision-making constitute a central dimension of group empowerment, reflecting organizational capacity and collective action grounded in shared learning processes and a sense of group belonging. In this regard, the selected studies describe psychosocial interventions that involve empowerment groups, but not necessarily empowered groups, since they do not always provide opportunities for action beyond the group, nor do they ensure continuity and autonomy over time once the psychosocial intervention ends. 

#### 3.4.2. Community-Level Empowerment

Community-level empowerment was observed in processes of civic participation and engagement with institutional actors (Abraczinskas & Zarrett, 2020; Forbes-Genade & van Niekerk, 2018; González et al., 2022; Montes et al., 2022; Moreno Álvarez, 2025; Wellman et al., 2025), as well as in shifts in recognition and legitimacy of young people as social actors (Dill, 2015; Trott et al., 2020; Wagaman et al., 2023; Scholtes-Bos et al., 2025; Wenitong-Schrieber et al., 2024).

##### Civic Participation, Co-Design, and Co-Governance

The selected studies show that action research, citizen science, and civic education initiatives constitute interventions in which young people engage in forms of participation that advance toward co-design and co-governance. In these experiences, participant groups negotiate with school and community authorities, while also formulating diagnoses, designing proposals, and contributing to decision-making in formal settings such as student councils, intersectoral working groups, or meetings with school leaders and municipal representatives. This reconfigures power relations, strengthens the influence capacity of youth collectives, and opens institutional channels for their agency. In this way, such interventions enable young people to act as knowledge producers, agents of change, and co-managers of local policies, fostering community empowerment through participation and influence (Abraczinskas & Zarrett, 2020; Forbes-Genade & van Niekerk, 2018; González et al., 2022; Montes et al., 2022; Moreno Álvarez, 2025; Wellman et al., 2025).

##### Transformation of Power Relations and Public Legitimacy

The studies analyzed show that community empowerment, in the case of youth groups, involves processes that go beyond formal participation and encompass transformations in power relations and public legitimacy. Young people move from consultative positions to decision-making roles, reflecting shifts in intergenerational hierarchies and in community governance mechanisms (Abraczinskas & Zarrett, 2020; González et al., 2022; Montes et al., 2022; Moreno Álvarez, 2025).

The legitimization of their expertise is expressed in their recognition as knowledge producers and situated actors who generate knowledge from their lived experiences, particularly in action research, community arts, and narrative production initiatives that enable them to challenge deficit-based representations and construct counter-narratives (Dill, 2015; Hillel Lavian, 2018; Trott et al., 2020; Case, 2017).

These processes are strengthened when their contributions gain social visibility, are incorporated into institutional decisions, or generate new spaces of interaction with authorities and community actors (Wagaman et al., 2023; Scholtes-Bos et al., 2025). In certain contexts, particularly those marked by structural inequalities or coloniality, community empowerment takes on an explicitly political and transformative character, reconfiguring cultural norms, institutional practices, and authority dynamics through forms of collective self-organization that assert rights, identities, and situated knowledge (Wenitong-Schrieber et al., 2024; Goodkind et al., 2020; Horwitz & Mares, 2025).

The transformation of power relations and public legitimacy constitutes a central dimension of youth community empowerment and is expressed through relational, symbolic, and structural transformations. These aspects—the transformation of power relations and the enhancement of public legitimacy—are crucial to the interaction and influence between youth groups and other groups in society, making them an indispensable feature of community-level youth empowerment processes.

## 4. Discussion

This exploratory review synthesised 32 empirical studies that examine youth empowerment among adolescents (primarily aged 10 to 18) and go beyond an exclusively individual perspective. The findings highlight the diversity of contexts, populations and intervention settings in which youth empowerment is promoted. This reinforces the idea that empowerment processes are deeply embedded in social, cultural and institutional environments. In line with previous research, these interventions are frequently implemented in contexts characterized by structural inequalities. Youth empowerment is closely linked to broader issues of social justice and inclusion. These findings should be interpreted with caution due to the predominance of qualitative designs, the limited availability of longitudinal data, and the inclusion of some studies with broader age ranges. Furthermore, while consistent with the exploratory nature of scoping reviews, the absence of a formal quality appraisal procedure may constrain the assessment of methodological robustness across studies. The diversity of implementation settings ranges from schools and community organisations to public and artistic spaces. Taken together, this reflects the multiple environments in which empowerment processes can emerge. Across these contexts, the consistent presence of adult actors underscores the central role of youth–adult relationships. The findings support the relevance of power-sharing dynamics as a core condition for meaningful youth empowerment. This aligns with the critical social theory of youth empowerment (Jennings et al., 2006).

From a methodological perspective, the predominance of qualitative approaches reflects the relational, contextual and process-oriented nature of empowerment. As proposed by Rappaport (1984) and Zimmerman (2000), empowerment is not a fixed outcome but a dynamic process shaped by interactions between individuals and their environments. Qualitative and participatory methodologies appear particularly well suited to capturing these dynamics. In contrast, quantitative approaches tend to operationalise empowerment in more individualised terms. This may limit the analysis of collective and structural processes.

The search strategy also reflects a deliberate theoretical boundary. By retaining empowerment as the organising construct and using the community-level criterion to refine the search, the review prioritised conceptual coherence over maximal thematic breadth. This means that some relevant studies using adjacent vocabularies, such as youth organizing, civic engagement or collective action, may not have been captured. This limitation should be considered when interpreting the comprehensiveness and representativeness of the review.

At the group level, the findings highlight the central role of relational processes in the construction of empowerment. The development of social support networks and a sense of belonging emerge as a foundational condition for collective agency. This reinforces the importance of safe and supportive environments. These findings are consistent with the critical social theory of youth empowerment (Jennings et al., 2006) and with the relational dimension of empowerment proposed by Christens (2012), which emphasises mutual support and collaborative processes.

In addition, group settings function as spaces for collective learning and meaning-making. In these spaces, young people reinterpret their experiences and develop critical understandings of social realities. These processes enable the transformation of individual concerns into shared social analyses. This contributes to the development of critical consciousness. Furthermore, group-level empowerment is expressed through collaborative action and participation in decision-making processes. This illustrates how young people develop organisational capacities and exercise collective agency within structured settings.

A critical distinction emerging from the findings concerns the difference between empowering groups and empowered groups or organisations (Zimmerman, 2000). While many of the interventions reviewed create conditions that support participation, collective learning and shared decision-making, fewer provide evidence that these groups achieve sustained collective agency or sociopolitical influence beyond the intervention context. In this sense, most initiatives appear to function as empowering settings for individuals rather than as empowered collective actors capable of producing change in their environments.

This limitation is closely linked to the issue of sustainability. Few studies provide evidence regarding the continuity of youth groups once the structured intervention ends. As a result, it remains unclear whether the collective processes described translate into stable organisational forms or dissolve once external facilitation is withdrawn. This points to the need to move beyond short-term intervention logics and to examine empowerment as a process that unfolds over time, requiring longitudinal approaches and sustained community integration.

A related tension concerns the degree of autonomy granted to young participants. Although many interventions promote participation and youth voice, these processes are frequently embedded within adult-mediated structures (Cargo et al., 2003; Diaz & Paceley, 2024; Mouchrek & Benson, 2023). This raises the question of whether young people are being supported to lead and sustain their own groups, or whether their participation remains contingent on externally driven programmes. The limited evidence on youth-led continuity suggests that empowerment may remain constrained if young people are not enabled to exercise leadership beyond the boundaries of the intervention.

At the community level, empowerment is primarily expressed through participation in civic processes and engagement with institutional actors. The findings suggest that youth empowerment extends beyond the group when young people are able to influence decision-making spaces and contribute to the design of community initiatives. In this context, empowerment involves shifts in recognition and legitimacy. Young people are increasingly acknowledged as social actors and knowledge producers. In settings characterised by structural inequalities, these processes may acquire a more explicitly political dimension. They can challenge dominant power relations and representations.

At the community level, these findings also point to the importance of inter-organisational dynamics. Empowerment cannot be understood solely within the boundaries of a single group. It must be analysed in relation to the broader ecosystem of organisations, institutions and actors. The extent to which youth groups are able to connect with, influence or become integrated into these networks appears to be a key condition for community-level empowerment. However, this dimension remains underexplored in the literature.

Despite these contributions, the review reveals several tensions within the field of youth empowerment research. First, focusing the research question on a specific theoretical concept—such as empowerment—while addressing specific needs within the field of community psychology, may have excluded from the eligible studies those that address other conceptualizations, such as civic engagement and citizenship, among others.

Second, the lack of standardized criteria in research involving children and adolescents regarding how to define age ranges within this population. This scoping review established an age range of 10 to 18 years as an inclusion criterion, given the aim to explore empowerment processes during school years with the intention of planning future interventions for this population. The selection followed this criterion; however, considering the characteristics of the field of study described previously, studies were included whose samples met the criterion but had a limited expansion of the age range. This makes it difficult to present data broken down by age and means these results should be interpreted with caution.

Third, there is a persistent predominance of an individualised perspective. Even when studies address group and community contexts, the evaluation of outcomes tends to focus on psychological dimensions such as self-efficacy or individual agency. As a result, collective processes are often conceptualised as mechanisms that facilitate individual empowerment rather than as forms of empowerment in their own right.

Fourth, the findings point to a lack of conceptual clarity. The term empowerment is frequently used interchangeably with related constructs such as participation, leadership or well-being. These terms are often used without a clear theoretical distinction. This ambiguity limits the capacity to analyse empowerment as a specific and multi-dimensional process.

Fifth, the extent of community-level empowerment varies considerably across studies. While some interventions report meaningful participation and influence in decision-making processes, others remain at a consultative or symbolic level. This variability suggests that the transition from participation to structural transformation remains a key challenge in youth empowerment practice.

Finally, the findings highlight the dependence of empowerment processes on institutional and intergenerational conditions. The sustainability of empowerment appears to rely on the willingness of adult actors and institutions to share power and create enabling environments. In contexts characterised by structural constraints, such as poverty or discrimination, the capacity of young people to influence their environment may be limited. This remains the case even when individual and group-level processes are strengthened.

Taken together, these findings suggest that, although the literature increasingly incorporates group and community perspectives, the development of these dimensions remains limited. This underscores the need to further advance both theoretical and empirical approaches that conceptualise empowerment as a multi-level process involving not only individuals, but also groups, organisations and communities. Advancing youth empowerment research requires a shift from analysing interventions as bounded experiences to examining the conditions under which collective actors emerge, sustain themselves, and influence their environments.

## 5. Conclusions

This scoping review synthesises empirical evidence on youth empowerment interventions involving adolescents aged 10 to 18 that extend beyond the individual level. The findings show that empowerment processes are closely linked to participatory, relational and action-oriented experiences in which young people engage collectively, develop shared understandings of social issues and connect with their communities.

The review highlights the importance of group and community dimensions in youth empowerment. Group environments provide conditions for mutual support, collective learning and collaborative action, while community engagement enables young people to participate in civic processes and gain recognition as social actors.

However, the findings also reveal a significant limitation in the current literature. Even when collective contexts are considered, the analysis tends to prioritise psychological outcomes, with groups and communities often treated as settings for individual empowerment rather than as empowered entities. As a result, there is still limited understanding of how collective and community empowerment processes are constructed and sustained.

Overall, the study underscores the need to advance youth empowerment research as a multi-level process that integrates individual, group and community dimensions.

### 5.1. Implications for Practice

In line with the findings of this review, the implications for practice vary according to the specific dimensions of empowerment identified at the group and community levels. The reviewed studies suggest that different relational, organisational and participatory processes are associated with distinct forms of youth empowerment. Consequently, implications for practice should not be formulated as generic recommendations for participation, but as context-sensitive strategies linked to the specific collective processes identified in the evidence.

First, the findings related to collaborative action and participation in decision-making suggest that youth empowerment is strengthened when young people engage in action-oriented practices with real influence over projects and collective processes (Abraczinskas & Zarrett, 2020; Cardarelli et al., 2021; Montes et al., 2022). This indicates that interventions should move beyond consultative participation and create concrete opportunities for young people to deliberate, negotiate priorities and co-design actions with visible consequences within their communities and organisations.

Second, it is essential to create conditions that position young people as protagonists. This includes participation in decision-making, in defining relevant issues, and in shaping intervention strategies. Such protagonism requires deliberate mechanisms for redistributing power between young people and adult actors. Without this, participation risks remaining symbolic.

Third, the findings concerning social support, belonging and collective meaning-making indicate that group-level empowerment depends on the development of stable relational processes rather than solely on individual participation. Studies describing mutual support networks, shared critical reflection and collective identity formation (Ellington et al., 2023; Goodkind et al., 2020; Forbes-Genade & van Niekerk, 2018) suggest that interventions should intentionally cultivate group cohesion, continuity of participation and spaces for shared interpretation of social experiences. These elements appear to function as foundational conditions for the emergence of collective agency.

Fourth, the findings at the community level suggest that empowerment processes expand when youth groups establish connections with institutional actors, civic spaces and community decision-making structures (González et al., 2022; Moreno Álvarez, 2025; Wellman et al., 2025). Interventions that facilitated interaction with schools, municipalities or community organisations showed greater potential to generate shifts in recognition, legitimacy and influence. This suggests that strengthening inter-organisational relationships and opportunities for civic engagement constitutes a key condition for moving from group-level empowerment toward community-level empowerment.

Beyond design and implementation, the findings point to the need to rethink how youth empowerment interventions are evaluated. Current evaluation practices tend to prioritise individual-level outcomes. This creates a misalignment between how empowerment is conceptualised and how it is measured.

Evaluation strategies should incorporate the analysis of groups as collective agents. This requires moving beyond individual indicators and examining how groups develop shared agency, organise collective action and sustain their activity over time. Group-level processes such as decision-making, mutual support, shared identity and collective learning should be treated as outcomes in their own right.

In addition, evaluation should consider community-level dynamics. This includes analysing how youth groups interact with other organisations, institutions and actors within their environment. The capacity to build alliances, influence decision-making spaces and become recognised as legitimate actors are key indicators of community-level empowerment.

A further implication concerns the need for longitudinal approaches. Empowerment processes unfold over time. Short-term evaluations are not sufficient to capture whether groups sustain themselves, evolve or disappear once interventions end. Longitudinal designs allow for the assessment of continuity, transformation and long-term impact.

Evaluating relational processes is also essential. This involves examining how relationships are built, maintained and transformed within groups and between groups and their environments. Relational dynamics such as trust, reciprocity and power-sharing are not secondary elements. They are core mechanisms of empowerment and should be explicitly assessed.

Finally, these implications suggest the need to expand existing evaluation frameworks. Both practice and research should incorporate indicators that capture collective and community dimensions of empowerment. This includes developing tools capable of analysing group behaviour, inter-organisational dynamics and ecosystem-level change.

Taken together, these implications call for a shift in practice. Youth empowerment interventions should not only aim to develop individual capacities. They should also support the emergence of collective actors and the transformation of community contexts. This requires aligning intervention design, implementation and evaluation within a coherent multi-level and relational framework.

At the same time, these implications should not be interpreted as universally applicable formulas. The reviewed studies show that empowerment processes are strongly shaped by contextual conditions, including institutional cultures, intergenerational relationships, structural inequalities and opportunities for civic participation. Consequently, the transferability of these implications depends on the extent to which interventions are able to adapt to the relational, organisational and sociopolitical characteristics of specific contexts. Rather than prescribing fixed models, the findings of this review support the importance of context-sensitive and ecologically grounded approaches to youth empowerment.

### 5.2. Future Directions

Building on these implications, future research on youth empowerment should place greater emphasis on the development of group and community dimensions, which remain underexplored in the current literature.

A key priority is to move beyond approaches that conceptualise groups and communities primarily as contexts for individual change. Future studies should examine them as units of empowerment in their own right. This includes identifying the characteristics of empowering and empowered groups or organisations, as well as the processes through which collective agency is constructed, sustained and transformed over time.

Further research is needed to better understand the conditions that enable youth groups to achieve continuity beyond intervention settings. This involves examining the factors that support or hinder the sustainability of collective processes once external facilitation is withdrawn. Particular attention should be given to the role of youth leadership and the degree of autonomy granted to participants in shaping and maintaining these groups.

Future studies should also explore how youth empowerment processes unfold within broader community ecosystems. This includes analysing how youth groups interact with institutions, organisations and other social actors, and the extent to which they are able to influence decision-making spaces and contribute to community-level change. Understanding these inter-organisational dynamics is essential for advancing knowledge on community-level empowerment.

There is also a need to advance methodological approaches capable of capturing empowerment as a multi-level and relational process. This requires developing and applying evaluation strategies that go beyond individual-level indicators. Future research should incorporate measures that assess collective agency, group functioning and community-level outcomes.

Longitudinal designs are particularly important in this regard. They allow for the analysis of how empowerment processes evolve over time, including the emergence, consolidation and potential decline of youth-led groups and initiatives. Such approaches are necessary to assess the durability and long-term impact of empowerment processes.

Finally, future research should further develop theoretical frameworks that integrate individual, group and community levels of analysis. Advancing a relational and ecosystem-based understanding of empowerment will strengthen both conceptual clarity and empirical research in the field. Future reviews could complement this approach by examining how adjacent fields, such as youth organizing, civic engagement and collective action, conceptualise processes that overlap with empowerment. Such work would help clarify the boundaries, intersections and differences between empowerment and related constructs.

## Figures and Tables

**Table 1:** Characteristics of the studies included in the scoping review (*n* = 32).

Authors & Year	Objectives	Design	Participants (Characteristics of Participating Adolescents)	Data & Analysis	Key Findings on Empowerment at the Group and Community Levels
(Abraczinskas & Zarrett, 2020)	Feasibility of YPAR (Youth participatory action research) and YPAR + PA (Physical activity)	Mixed methods	64 students (female = 41) aged 11–15. 94% non-Hispanic Black/African American, 75% free/reduced lunch in the southeastern United States.	Youth Empowerment Scale (Quan) + journals, surveys, photovoice, interviews (Qual). Triangulation.	Youth shared decision-making; systems-level change requires a stakeholder support at multiple levels. YPAR + PA increased influence. Youth are viewed as experts from within, whose perspectives can bring about changes that “top-down” approaches often overlook.
(Bastable et al., 2023)	Evaluate Changing the Story, Leadership Development Programme within the Youth Accountability and Deaf Inclusion in South Africa	Q-method, a participatory research methodology.	38 youth (12–21 years of age) participated in the programme from a safe park (*n* = 23 vulnerable youth) and a special needs school (*n* = 15 Deaf youth).	Longitudinal Q-sort methodology to measure the youths’ changes in perceptions.	Youth gained insight into themselves not only as individuals, but as individuals who could guide others and contribute to their communities.
(Brown et al., 2015)	Compare youth partnerships (Paso del Norte Health Foundation and focused on tobacco control) with that of adults in health promotion.	Quantitative. Cross-sectional comparative study.	Youth participants (*n* = 44) were predominantly Hispanic, 45% female, and had an average age of 13 years.	Multidimensional battery of 10 partnership functioning constructs.	Youth–adult partnerships operate comparably to adult partnerships, offering young people substantive opportunities for agency, including skill development, relationship building, meaningful contribution, and engagement in change-oriented actions that foster civic identity. Young people face heightened participation barriers; however, these can be reduced through organizational adaptations that promote inclusion and strengthen advocacy capacity.
(Buelens et al., 2017)	Sufficient conditions necessary to foster human capital development among disadvantaged adolescents through volunteering in community sport.	Phenomenological Analysis (IPA) approach was used across eight community sport programmes within the “Street Action” initiative in Flanders.	Adolescents: 26 disadvantaged young people (M = 13.8; SD = 2.14), participating in four focus groups.	Semi-structured, in-depth interviews, Focus groups, Observations, Documentary analysis, Round-table discussion with sport partners. Inductive analysis.	These processes are associated with personal and interpersonal competencies. Their alignment with critical youth empowerment frameworks is only partial. Most programs do not reach the more transformative dimensions of critical empowerment.
(Cardarelli et al., 2021)	Critical components of authentic youth engagement in environmental health promotion, science communication, advocacy and research.	Comparative case study analysis of four initiatives on youth engagement to promote environmental education in rural (Central Appalachia) and urban (Cincinnati, Ohio) communities, using the Youth Empowerment Solutions (YES!) framework.	1. Mountain Air Youth Photovoice Project (10 participants aged 13–18). 2. Citizen Science Program (over 100 high school students) 3. Medical Camp (24 participants per session, 5th-8th grade urban youth) 4. Youth Tobacco Advocacy Training Program (20 youth in development phase; 80 youth in implementation phase). Workshop: 60 participants.	Categorization of case study elements using the Youth Empowerment Solutions (YES!)	Youth empowerment as a multilevel construct. Youth demonstrated agency to influence environmental health issues through real-world engagement; however, action-oriented opportunities remained comparatively scarce. Authentic youth engagement is framed as essential, requiring meaningful leadership roles, transparent decision-making processes, and institutional recognition of youth voice. The proposed model positions empowerment as an intergenerational, collaborative process. Barriers operate across individual, organizational and community levels.
(Case, 2017)	Propose a model of intervening that integrates positive youth development (PYD) and critical theory to address the social-structural inequalities faced by minority youths from low-income urban communities (LUC).	Ethnographic study (9 months).	Youth participants: 9 African American youths (mean age 18) involved in Peer Ambassadors (PA) in Champaign, Illinois.	Observations, Semi-structured interviews, Fieldnotes documenting observations. Thematic analysis.	Youth empowerment at the interpersonal and community levels was reported through their enactment of highly visible, responsibility-laden roles which expanded their perceived agency and civic influence. These roles fostered recognition as legitimate social actors. The program functioned as a counterspace that replaced deficit-oriented labels with a collective identity grounded in public engagement, enabling the emergence of counternarratives affirming youths’ value and civic potential. Supportive peer relationships further operated as structured systems of care. Community engagement enabling youth to develop civic competencies, navigate community systems, and form identities. Empowerment described as relational, collective, civic, identity-based, and action-oriented aligning with critical perspectives.
(Cicognani et al., 2015)	Organizational membership effects in different types of community and youth organizations on young people’s Social well-being.	Cross-sectional quantitative study.	835 adolescents and young adults. Age: 16–26 years. Adolescents (16–19): 38.1%. Gender: 50.4% female. From Emilia Romagna region (North Italy).	Structured questionnaire Included descriptive analyses, ANOVA, hierarchical regression, and bootstrapped mediation models.	Membership in organizations consistently strengthens young people’s empowerment (whether through occasional or ongoing participation). Empowerment functions as a central mediating mechanism between organizational participation and social well-being. Youth empowerment emerges within relational and participatory contexts that support youth voice, shared decision-making, opportunities to enact agency, and supportive organizational relationships.
(Dill, 2015)	Detail how youth engaged in participatory narrative analysis and asserted themselves as expert researchers and storytellers of their neighborhood.	Ethnographic study evolving into critical ethnography and youth participatory action research (YPAR), incorporating research poetry and interpretive poetry as methodological innovations.	25 African American and Latino youth, aged 12–20, recruited from multiple programs in East Oakland.	Semi-structured interviews, Participant observation for 8 months, Poetry workshops. Analysis occurred collaboratively, culminating in a poetry anthology.	Youth empowerment is enacted through their positioning as co-researchers. Participatory narrative and poetic methods further strengthen youth narrative agencies, enabling them to contest deficit-based portrayals, articulate complex socio-emotional experiences, and reclaim representational power. Public dissemination of poetic work expands their civic agency. Collective empowerment emerges through research collective, which facilitates shared reflection, mutual support, and the co-construction of multi-layered identities. Spirituality serves as a framework for coping and seeking meaning that young people use to interpret violence, define their aspirations, and express their will to survive. Artistic creation operates as an empowerment practice that transforms lived disorder into expressive, communal, and political action.
(Ellington et al., 2023)	Document how a local community organization (Salud y Cariño) supported youth during and after the COVID-19 through two youth-led YPAR projects in Live Oak, in Santa Cruz County, California (U.S.).	A qualitative, community-engaged YPAR design.	Approximately 25 youth (students of color, the majority Latinx, including several identifying as non-binary or transgender). The documentary included 7th–12th graders; the photovoice project involved 8th graders.	Field notes, artifacts and youth outputs were compiled as data sources. Open coding; Extended case method; Deductive application of theories on YPAR, youth voice, and community engagement; and Inductive/grounded theory elements.	Salud y Cariño functioned as a stable, relational support system, described as a “second family”, that fostered continuity, mentoring, and social connection during periods of isolation. The program created a welcoming and safe interpersonal environment that validated diverse gender identities, facilitated emotional safety, and reduced stress. Everyday activities infused with care strengthened emotional resilience and reinforced youths’ sense of agency. At the interpersonal and community levels, empowerment emerged through youth-led decision-making, opportunities to construct and share their own narratives of inequity, and participation in public dissemination spaces. The program also promoted reciprocal empowerment, as adults acknowledged gaining purpose and insight from youth leadership. Cultural empowerment was central emphasized. Latinx histories, communal values, identity pride, and resistance while explicitly affirming gender diversity within Latinx cultural contexts.
(Farahzadi et al., 2025)	Investigate the feasibility and acceptability of an Assertive Community Treatment (ACT) intervention for Afghan migrant adolescents in Tehran.	Quantitative, semi-experimental pre-test/post-test design	11 Afghan migrant adolescents, aged 13–15, residing in a low-income neighborhood.	Quant: Demographic questionnaireEcomaps; HITS tool (domestic violence screening); Illinois Bully Scale. Qual: Semi-structured interviews; Home visits, Observational reports; and Detailed case reports. Wilcoxon test for pre/post changes.	Although it does not explicitly frame its findings through empowerment, it documents outcomes aligned with interpersonal and community-level youth empowerment processes. The intervention strengthened adolescents’ social integration by expanding peer networks and reinforcing their sense of belonging in school and community settings. Participants increased their engagement in collective activities, suggesting greater agency in accessing communal spaces and improved confidence in social environments. The program also enhanced family linkages to support organizations and increased adolescents’ contact with trusted adults.
(Favre et al., 2025)	Strengthen the resilient development of youth who have experienced parental physical violence by involving them directly in the creation of educational video materials.	Qualitative, participatory, design-based approach.	119 adolescents (ages 12–20) recruitment through schools and SEMO programs (socioeconomically disadvantaged or vulnerable youth) in German- and French-speaking regions of Switzerland. From these, 12 adolescents (ages 12–16) voluntarily formed expert groups	Six half-day workshops. Iterative co-creation cycles.	Youth empowerment is depicted as a relational and collective process rooted in participatory co-creation, where adolescents hold narrative and creative authority. Empowerment emerges through interdependence within family, school, and peer systems, highlighting co-learning and access to supportive conditions. Help-seeking is reframed as an expression of agency, while recovery from trauma is conceptualized as reclaiming identity and capacity for action. Narrative and metaphor-based problem-solving further illustrate empowerment as the activation of resources, negotiated access to support, and collaborative challenge-solving. Authentic representation, inclusion, and the production of tools for peers underscore empowerment as a collective, future-oriented practice.
(Ferrer-Fons et al., 2022)	Non-formal arts education attenuates socioeconomic and cultural barriers in a vulnerable context.	Extreme case study strategy.	10 Young people aged 16–21 (migrants, second generation, mono-parental families, low-qualified or unemployed parents) from “Raval” neighborhood in Barcelona (Spain).	Non-participant observation, semi-structured qualitative interviews. Thematic and inductive coding.	Youth empowerment emerges through emotionally and creatively engaged arts-based participation, which enables access to personal and collective experiences. Empowering conditions are grounded in non-hierarchical, trust-based educator-youth relationships. At the interpersonal level, empowerment is associated with enhanced socio-emotional competencies. Collectively, empowerment is fostered through collaborative artistic practices emphasizing cooperation, shared decision-making, and mutual support. Community-level empowerment is further expressed through the development of critical environmental awareness and an understanding of culture as a tool for social transformation. Structural constraints limit empowerment.
(Forbes-Genade & van Niekerk, 2018)	Contributions of the Girls in Risk Reduction Leadership (GIRRL) Program in building resilient communities through Participatory Action Research (PAR)	PAR intervention and study.	21 adolescent girls, ages 13–18, from Sonderwater, an informal settlement in South Africa, characterized by poverty, social exclusion, high disaster risk, and multiple social stressors. Girls were selected based on vulnerability criteria.	Focus group discussions. Participatory tools. Narratives of life. Journals. Iterative, reflective analysis embedded in PAR cycles	Youth empowerment at group and community levels is reported as multifaceted, encompassing agency, collective action, and sociopolitical influence. Emerges through adolescents’ participation in decision-making structures, where disadvantaged girls exercise agency within community governance and risk-reduction processes. It is further reflected in their assumption of leadership roles and increased public visibility. Programs also emphasize the development of social capital by fostering partnerships between girls and municipal actors, enabling intergenerational role shifts, and generating broader stakeholder recognition of girls’ contributions. Skill-building is framed as preparation for collective action. Empowerment additionally involves the cultivation of critical awareness. Collective identity, solidarity, and mutual support are highlighted as relational dimensions of empowerment. Youth influence is described as contributing to cultural and institutional change.
(Godfrey & Grayman, 2014)	Examine whether an open classroom climate predicts youth’s critical consciousness, exploring differences between young people from racial or ethnic minority and majority groups.	Quantitative, multilevel analysis. Data from the 1999 IEA Civic Education Study, a nationally representative cross-sectional survey of U.S. ninth-graders.	2774 U.S. ninth-graders, average age 14. 50% female; 58% white; remainder represented multiple racial/ethnic minority groups.	Secondary analysis. Two-level regression models. Tests of moderation by racial/ethnic majority versus minority status	Open classroom climates function as empowering institutional contexts that enhance youths’ sociopolitical efficacy and support their engagement in community-oriented critical action. The empowering effects vary by racial and ethnic background in school settings; however, these inclusive environments appear to offer forms of structural empowerment that can mitigate marginalization.
(González et al., 2022)	To engage and empower students from schools in Bogotá, Colombia to use the Our Voice model to assess and seek to improve their local school environments	Mixed-methods design within the Our Voice “citizen science by the people” participatory action.	97 student citizen scientists. Ages: 9–18 years (mean 13.4). From 5 public schools in Bogotá. Majority from low socioeconomic strata; over half female.	Surveys, Environmental Assessments with the Discovery Tool App. Thematic analysis.	Youth empowerment is reported through multiple interrelated processes that enhance agency. These include the development of youth as “agents of change” through active participation in institutional decision-making and civic advocacy. Empowerment also emerges from collective reflection and shared responsibility for the school environment. At the community level, strengthened and sustained civic engagement is framed as a long-term capacity aligned with broader youth empowerment models. Despite structural limitations, young people influenced institutional priorities, signaling partial but significant shifts in power dynamics.
(Goodkind et al., 2020)	Contrast traditional (individual) and alternative (collective) models of resilience within the context of an empowerment program for Black girls.	Mixed-methods evaluation of an empowerment program.	33 Black high-school girls in Allegheny County, Pennsylvania (13–17 years old), attended a mix of public and charter schools; some were U.S.-born and others recently immigrated from African countries.	Quan: Online surveys. Descriptive statistics, correlations, *t*-tests. Qual: Participant observation. 16 Individual interviews and a focus group (*n* = 13). Deductive coding.	The program enhanced critical reflection by shifting girls’ interpretations of personal experiences toward structural analyses of racism, sexism, and adultification, alongside a rejection of neoliberal individualism. Participants developed a positive gendered racial identity. Interpersonally, empowerment manifested through mutual support and communal validation, reframing well-being as interdependent. At the community level, empowerment was articulated as the convergence of critical consciousness, collective identity, and collective action.
(Hillel Lavian, 2018)	Accompany a group of teenagers in risk situations through a photography workshop as part of a personal and social empowerment project.	Radical feminist qualitative research design applying Photovoice methodology.	7 teenagers, ages 14–18, living in a shelter for youth at risk in central Israel. One boy and six girls.	Photographs taken by participants. Documented conversations and discussions about the photograph. Researcher’s diary. Interactions in a closed Facebook group. Holistic content analysis and Interpretive content analysis of photographs and narratives. Coding of themes emerging during discussions.	Youth empowerment is depicted as a relational and collective process in which visibility functions as a key mechanism. Participatory photography fosters critical awareness and open dialogue on complex social issues. Individually, empowerment appears through increased agency, self-recognition, and narrative reclamation. At the community level, shared reflection strengthens collective identity and mutual support.
(Horwitz & Mares, 2025)	Explore the developmental outcomes of five youth food justice (YFJ) programs in the Northeastern U.S.	Comparative qualitative case study of program Grounded in Positive Youth Development (PYD).	17 participants: 11 youth, aged 15–20; self-identified as Hispanic, Black/African American, Black/Caribbean, White, Cape Verdean, African, and Puerto Rican.	Open-ended, semi-structured interviews. Initial coding, axial coding and condensation into broader themes.	Youth empowerment was described across interrelated interpersonal, group, and community dimensions. Critical consciousness developed as youth recognized systemic oppression. Supportive relationships with adult mentors and access to safe, inclusive program spaces enhanced emotional security and personal growth. Community engagement fostered collective agency, civic participation, and socially valued roles, strengthening community-rooted identities.
(Lasczik et al., 2025)	Explore how engaging in a/r/tography can empower artivist childhoods.	A child-framed participatory a/r/tographic inquiry, combining Arts-based research, walking/mapping practices, and collaborative data creation and analysis.	Young people (under 18) enrolled in a Special Assistance Secondary School in Southeast Queensland; youth identified as at risk of disengagement from schooling.	Walking-based exploration, visual mapping, and various individual and collaborative artmaking practices. Youth conducted an initial rhizoanalysis and. Analyses were both collective and individual and developed rhizomatically.	Arts-rich, socially just pedagogies fostered interpersonal and collective empowerment by enhancing political awareness, critical literacy, and public expressive skills. A/r/tographic practices supported identity transformation and new relational orientations. Youth also influenced institutional structures by co-designing curriculum, with their contributions adopted at the school level, evidence of structural, community-level empowerment.
(Massó Guijarro, 2018)	Explore the potential of performing arts, specifically theatre, as a socio-educational tool in challenging contexts.	Qualitative research within a hermeneutic paradigm using: ethnographic approach and participatory observation.	Young people participating in a theater workshop at a socio-educational center (Córdoba, Argentina). Mostly male, 12–21 years old, low socioeconomic status and a range of other experiences of vulnerability.	Participatory observation conducted throughout the theater workshop. Field notes. Grounded Theory principles. Hermeneutical interpretation	Youth empowerment as resistance to oppressive structures with theater. Horizontal and dialogic relations position them as active agents, while participatory artistic practices validate their capacities and recast diversity as a collective asset. Creative production challenges cultural hierarchies and fosters expressive agency, countering symbolic violence. Interventions that destabilize traditional educational hierarchies reinforce inclusion and collective empowerment.
(McKay et al., 2022)	Outcomes of Intimate Partner Violence/Teen Dating Violence (IPV/TDV) victimization screening and universal education other than disclosure	Mixed-methods randomized field test	Youth (*n* = 648). English-speaking, ages 13+; racially/ethnically diverse. Additional interviews with 8 youth participants	Quantitative surveys. Inductive qualitative analysis of interviews. Post-intervention surveys. Descriptive stats; OLS and logistic regression; Bonferroni correction.	Interventions promoted youth empowerment by triggering reflection on relationship norms, clarifying unhealthy dynamics, and increasing awareness of safety resources. Empowerment was further supported by staff interactions marked by authenticity and respect, which enhanced youths’ sense of help-seeking ability. Program environments fostering safety, trust, and belonging enabled youth to consider protective options. At a broader level, organizational improvements, indirectly reinforced empowerment by strengthening the surrounding care ecosystem.
(Monteiro et al., 2015)	Culture Circles (CC) as a health education intervention involving adolescents in the collective construction of knowledge about strategies to prevent violence.	Participatory action research with a qualitative approach, based on Paulo Freire’s pedagogy of CC.	11 adolescents (6 girls, 5 boys), aged 15–19, enrolled in public secondary education in a highly vulnerable community in Recife (Brazil).	Participant observation; Field diary notes; Photographic records; Audio recordings; and Projective techniques. Qualitative triangulation of data. Critical-dialectical interpretation.	Youth empowerment is portrayed as a collective, relational process that strengthens critical consciousness, sociopolitical agency, and adolescents’ roles as community change agents. Through dialogical practices, creative expression, and intersectoral networking, adolescents co-developed strategies for community transformation while enhancing autonomy, belonging, and collective efficacy.
(Montes et al., 2022)	Valuate the impacts of the Our Voice citizen science method among adolescents in Santa Ana (Barú, Colombia).	Descriptive, first-generation formative pilot study using the Our Voice “by-the-people” citizen science methodology.	11 adolescent citizen scientists, aged 13–17, members of the school’s science club (7 girls, 3 boys completed all activities).	Technology-enabled community walks using the Stanford Discovery Tool to collect geotagged photos, audio/text comments, and ratings of school environment features. Pre-walk survey. Participatory mapping workshops. Topics classified into themes.	The project demonstrated youth empowerment through increased agency, collective action, and civic engagement. Participatory methodologies enabled adolescents to assume leadership roles, influence institutional decisions, and shift local power dynamics. Youth-led advocacy secured concrete commitments from school authorities, while project activities expanded dialogue and coordination across the wider community.
(Moreno Álvarez, 2025)	Analyse the implementation and impact of the S^2^Cities programme in Envigado, Colombia.	A qualitative case study grounded in the methodological approach of the S^2^Cities Fellowship Programme.	More than 90 young people across three cohorts of the programme. Young participants designed and implemented 47 pilot initiatives.	Monitoring and evaluation data from the programme’s internal measurement systems Qual: Youth testimonies, Documentation of initiatives, and Records of alliances and advocacy processes. Analysis organized across individual, relational and structural levels.	Youth empowerment is reported as the collective strengthening of agency in local governance and public-space management. Empowerment emerges through high-trust, cross-sector networks that expand relational capacity and enable coordinated, system-level action. At the community level, empowerment is reflected in youths’ ability to mobilise resources, sustain initiatives beyond programme cycles, and shape formal policy mechanisms and territorial planning. It is also expressed through the collective reclaiming of public space, redefining cultural practices, and fostering social cohesion via intergenerational dialogue, community mobilisation, and violence-prevention efforts.
(Ruhr & Fowler, 2022)	Explore whether the experiences, perceptions, and perceived benefits voiced by underserved minority youth in a Southern U.S. school district.	A qualitative evaluation using focus groups.	48 youth, ages 7–19, 46% female. 34 caregivers. Youth who had participated in one or more of three empowerment programs, came from economically disadvantaged backgrounds.	Separate in-person focus groups with youth and caregivers. Semi-structured interview. Note-taking. A theory-driven codebook based on the Five C’s and Big Three. Thematic analysis.	Youth empowerment as a multi-level process grounded in competence development, life-skill acquisition, and meaningful sociocommunity participation. Enhanced agency, communication, and self-expression strengthened youths’ effectiveness in interpersonal and group contexts. Independent decision-making, leadership practice, and adaptive problem-solving further supported functional autonomy. Opportunities to lead community initiatives enabled authentic youth voice and influence. Contributing to community improvement fostered collective efficacy and reinforced internal growth. Safe, inclusive program environments enhanced belonging, trust, and readiness for leadership. Reported gains included increased confidence, ethical agency, and adaptive functioning. Supportive, consistent adult–youth relationships provided essential scaffolding.
(Scholtes-Bos et al., 2025)	Explore the impact of the Food Boost Challenge (FBC) beyond changes in healthy eating in Netherlands.	A community-up participatory action research (PAR) design, implemented across two 9-month Food Boost Challenges. Mixed-methods design aligned with Community-Based Participatory Research (CBPR) indicators.	Young people aged 10–24. Stakeholders.	Semi-structured interviews with participants. Digital feedback surveys. Informal partner-management discussions. Peer-research data. Findings were grouped into descriptive theme.	Youth empowerment emerged through increased agency, emotional investment, and meaningful participation in food-system change. Reciprocal learning and reduced hierarchies strengthened youths’ confidence, skills, and legitimacy within multi-stakeholder settings. Capacity building and public recognition enhanced their perceived competence and visibility, while community-level collaboration expanded collective networks. Positioning young people as co-creators of feasible solutions also exposed gaps between policy ideals and lived realities, reinforcing empowerment through pragmatic engagement.
(To et al., 2020)	Association between youth empowerment in the community and young people’s creative self-efficacy (CSE) and between youth–adult partnerships (YAPs) and CSE.	Quantitative, cross-sectional survey.	2653 young people aged 11–24, recruited from 16 Integrated Children and Youth Services Centres (ICYSCs) across Hong Kong. Nearly equal proportions of males and females and represented diverse education levels.	Self-administered questionnaire. Descriptive statistics of demographic variables. Pearson correlations. Hierarchical regression analyses.	Community-level youth empowerment, via voice, shared decision-making, and meaningful community roles. YAP defined by reciprocity and authentic collaboration, operates as empowering relational structures. Empowerment is framed as a structural and relational construct that disrupts power imbalances and position. Such contexts strengthen young people’s belief in their capacity to innovate and require ongoing attention to power dynamics and reflexive practice.
(Trott et al., 2020)	To explore processes of individual and collective empowerment among youth participating in an integrated art-science course (“Photo-Environment”) in Jacmel, Haiti.	Qualitative study within a participatory action research (PAR) framework.	21 students (children, adolescents, and two adults) took part in the Photo-Environment course. Interview participants: 5 youth (female: 4; ages 8–17).	Semi-structured interviews with students and staff. Thematic analysis.	Youth engagement in arts-based initiatives enabled them to contest deficit-driven narratives about Haiti and to reframe how their communities are represented locally and globally. Through the Photo-Environment program, participants challenged cultural norms that position youth as unable to question authority. Collectively, they acted as environmental advocates who influenced community members, decision makers, and international audiences. Individually, fostered critical awareness promoted youth-led environmental action, and supported the envisioning of alternative socio-environmental futures for Haiti.
(Wagaman et al., 2023)	Identify components of youth empowerment rooted in the experiences of young people engaged in Youth Participatory Action Research (YPAR) in a mid-size Mid-Atlantic urban community (one of the cities with the highest eviction rates in the US.)	Interpretive qualitative design.	15 youth and young adult researchers (ages 16–25) involved in three YPAR teams. 9 participated in the post-project focus groups. All focus-group participants identified as Black or African American.	Post-project focus groups. Observation tools Thematic analysis. Empowerment theory provided the initial coding structure.	Youth empowerment was depicted as an iterative, relational process encompassing confidence in one’s own expertise, its active mobilization in interpersonal and research contexts, the development of critical structural awareness, and the formation of collective ties grounded in shared experience. External recognition of youth as legitimate contributors to community change was also central. Empowerment depended on trust, accountability, shared expertise, personal relevance of the issue, and was strengthened through YPAR’s role in rebuilding collective capacity amid housing instability.
(Wellman et al., 2025)	In-depth assessment of a youth, climate empowerment program, called Climate READY—Climate Resilience Education and Action for Dedicated Youth.	Design and Development Research model. Mixed-methods evaluation. Implementation of curriculum.	Climate READY Ambassadors (CRAs): High school students ages 15–17. After-school youth: Elementary students ages 9–11.	Pre/post questionnaire. After-school pre/post surveys. Community post-event surveys. Quan: Matched-pair Student’s *t*-tests and Kolmogorov–Smirnov tests for non-normal distributions (CRAs). Independent *t*-tests for after-school learners. Significance threshold *p* < 0.05. Qual: Kernel analysis and thematic summarization.	Youth empowerment is portrayed as the development of civic capacity, community attachment, and socially recognized leadership. Through involvement in resilience planning, public engagement, and interaction with local authorities, young people gained legitimacy in climate governance. They assumed mentoring roles, received community validation as knowledgeable contributors, and developed the agency and skills needed to influence local change.
(Wenitong-Schrieber et al., 2024)	Provide a detailed case study of the establishment of a youth-led, community-based First Nations empowerment organisation, Deadly Inspiring Youth Doing Good (DIYDG), created by Aboriginal and Torres Strait Islander young people.	In-depth case study.	Young people: 5 participants aged 16–20. All participants hold tribal links to diverse Aboriginal and Torres Strait Islander groups in Queensland.	Group and individual interviews. Speeches, social media posts, promotional videos. Focus groups. Website and social media content. Meeting minutes, strategic documents, business planning canvasses. Observations. Analysed, and further articulated through iterative discussions among co-authors.	Youth empowerment is depicted as a collective, culturally grounded process of resistance and community self-determination. It is relational and rooted in First Nations knowledge, with group formation serving as a reclaiming of agency and narrative control. Decision-making is youth-led, while Elders provide non-authoritarian cultural guidance. Empowerment is enacted through a continuous, non-linear process integrating inspiration, capacity-building, and action. It is collective and intergenerational, oriented toward healing, cultural continuity, and future reclamation. Structural constraints position empowerment as inherently political, requiring sustained negotiation and resistance.
(Zuma et al., 2022)	Understand experiences and perceptions of the multilevel DREAMS (Determined, Resilient, Empowered, AIDS-free, Mentored and Safe) HIV prevention intervention for adolescent girls and young women (AGYW) in rural KwaZulu-Natal (South Africa).	Ethnographic qualitative study.	AGYW and ABYM (adolescent boys and young men) targeted by DREAMS (ages 10–24 for AGYW; 12–35 for ABYM).	Group discussions. Longitudinal in-depth interviews (IDI) with young people. IDIs with implementing partners and stakeholders. Community mapping and observations using structured checklists. Open coding followed by thematic refinement. Comparison of themes.	The study does not explicitly define empowerment, yet it illustrates interconnected individual, relational, community, and structural dynamics shaping AGYW’s agency. Individual gains in information and service access were weakened by restrictive gender norms and family-level disempowerment. Community-level empowerment emerged through safe spaces, mentoring, and mobilization in schools and dialogues, though uneven implementation and the exclusion of key groups limited broader collective impact. Structural efforts at multi-sectoral coordination showed partial promise.

## Data Availability

No new data were created or analyzed in this study.
